# The role of mesenchymal stem cell-derived EVs in diabetic wound healing

**DOI:** 10.3389/fimmu.2023.1136098

**Published:** 2023-02-28

**Authors:** Min Jiang, Xupin Jiang, Hongmei Li, Can Zhang, Ze Zhang, Chao Wu, Junhui Zhang, Jiongyu Hu, Jiaping Zhang

**Affiliations:** ^1^ Department of Plastic Surgery, State Key Laboratory of Trauma, Burns and Combined Injury, Southwest Hospital, The Third Military Medical University (Army Medical University), Chongqing, China; ^2^ Department of Oncology and Southwest Cancer Center, Southwest Hospital, The Third Military Medical University (Army Medical University), Chongqing, China; ^3^ Department of Geriatic Oncology, Department of Palliative Care, Department of Clinical Nutrition, Chongqing University Cancer Hospital, Chongqing, China; ^4^ Endocrinology Department, State Key Laboratory of Trauma, Burns and Combined Injury, Southwest Hospital, The Third Military Medical University (Army Medical University), Chongqing, China

**Keywords:** angiogenesis, mesenchymal stem cells, exosomes, diabetes wound, extracellular vesicles

## Abstract

Diabetic foot is one of the most common complications of diabetes, requiring repeated surgical interventions and leading to amputation. In the absence of effective drugs, new treatments need to be explored. Previous studies have found that stem cell transplantation can promote the healing of chronic diabetic wounds. However, safety issues have limited the clinical application of this technique. Recently, the performance of mesenchymal stem cells after transplantation has been increasingly attributed to their production of exocrine functional derivatives such as extracellular vesicles (EVs), cytokines, and cell-conditioned media. EVs contain a variety of cellular molecules, including RNA, DNA and proteins, which facilitate the exchange of information between cells. EVs have several advantages over parental stem cells, including a high safety profile, no immune response, fewer ethical concerns, and a reduced likelihood of embolism formation and carcinogenesis. In this paper, we summarize the current knowledge of mesenchymal stem cell-derived EVs in accelerating diabetic wound healing, as well as their potential clinic applications.

## Introduction

Diabetes mellitus is a group of chronic metabolic diseases characterized by chronic hyperglycemia and increasing incidences, which has become one of the serious threats to human health. Thus, the complications caused by diabetes should not be ignored, which result in high death and disability rate. Refractory skin wound or spontaneous skin ulcer is a common chronic complication of diabetes mellitus, represented by diabetic foot (1). Diabetic foot, also known as diabetic acromelic gangrene, refers to the lower extremity skin ulcer, infection or deep tissue destruction caused by neuropathy and peripheral vascular disease, and many patients are disabled because of amputation. This not only causes great mental pressure to patients, but also brings heavy economic burden. Therefore, it is necessary to explore a series of effective strategies to accelerate the healing of diabetic skin ulcers or wounds (2).

Diabetic wounds are characterized by chronic inflammation, impaired angiogenesis, and delayed re-epithelialization (3). Therefore, it is urgent to inhibit inflammation, and promote cell differentiation, angiogenesis, extracellular matrix (ECM) production, growth factor production and wound contraction in diabetic wounds. Stem cell transplants, due to their ability to self-renew and differentiate, can provide effective treatments. However, safety concerns have limited the clinical application of this technology (4). Therefore, it is indispensable to develop safe and effective therapeutic strategies based on stem cells. Recently, the performance of stem cells after transplantation has been found being increasingly attributed to their production of exocrine functional derivatives such as extracellular vesicles (EVs), cytokines, and cell-conditioned media (5). EVs contain a variety of cellular molecules, including RNA, DNA and proteins, which facilitate the exchange of information between cells. EVs have several advantages over parental stem cells, including a high-profile safety, no immune response, fewer ethical concerns, and a reduced likelihood of embolism formation and carcinogenesis (6). At present, it has been reported that stem cell-derived EVs can participate in the regulation of cellular functions in wound healing of diabetic ulcers, promote neovascularization, stimulate collagen deposition, inhibit inflammation, and thus accelerate wound healing.

Hydrogel dressings are an emerging area for wound care, as they have good water retention, biocompatibility and biodegradability (7). They retain water in the wound and bind well to exosomes. The combination of exosomes and hydrogels can synergistically promote diabetic wound healing, which provides a new way for the application of stem cell-derived exosomes in chronic wound healing.

This review focuses on the mechanism by which mesenchymal stem cell-derived EVs inhibit inflammation, promote angiogenesis and accelerate re-epithelialization and collagen deposition in diabetic ulcers and the research progress in the combination of hydrogels to promote their future clinical application.

## Disorders in diabetic wound healing

**Figure 1:**
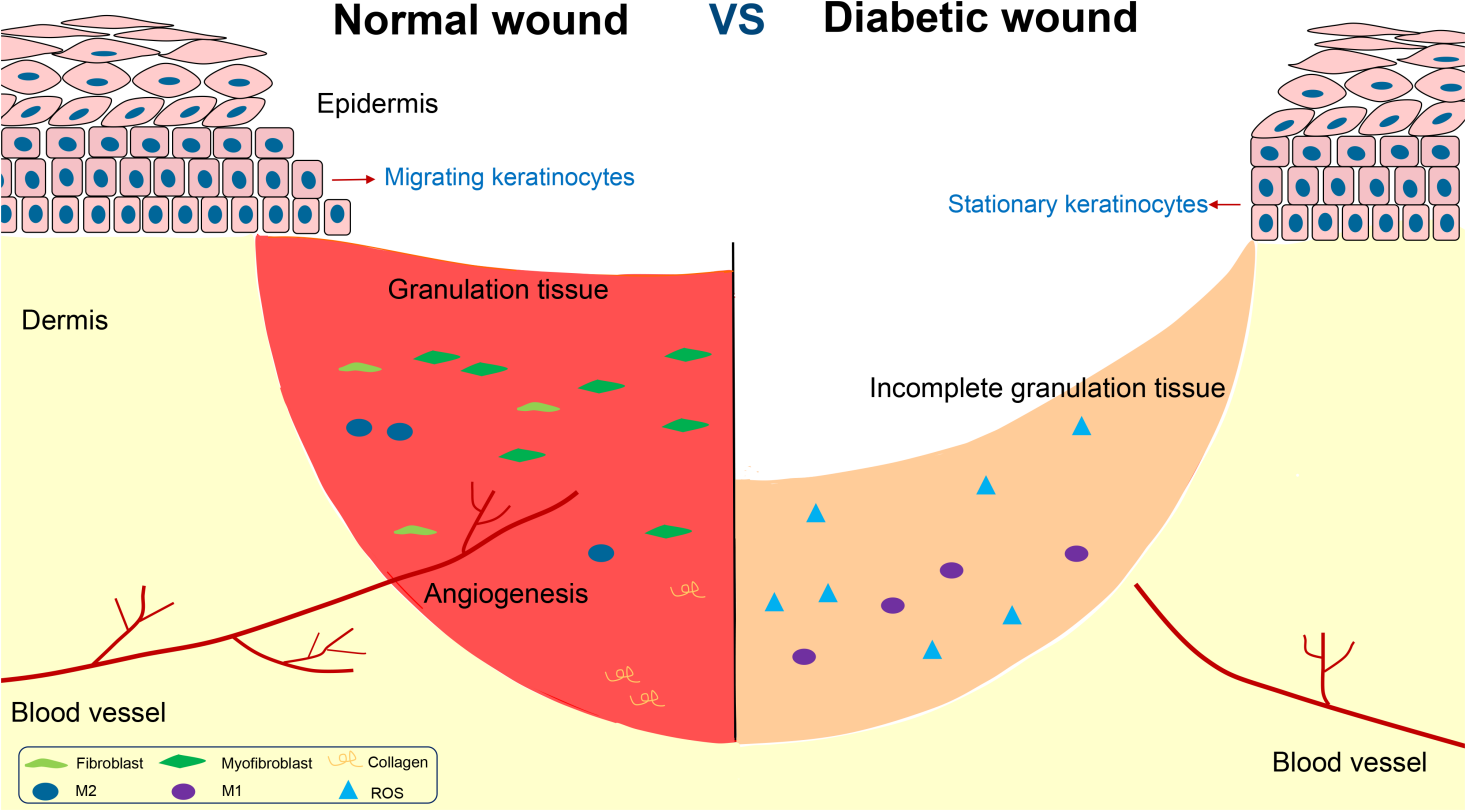
The disorders in the repair of chronic diabetic wounds.

Impaired wound healing is one of the main complications of diabetes mellitus, and diabetic foot is the main representative. Diabetic wound is featured by chronic inflammation, impaired angiogenesis, and delayed re-epithelialization and collagen deposition (**Figure 1**).

### Chronic inflammation

Diabetic wound is characterized by a chronic low-grade and prolonged inflammatory state, which is not conducive to wound healing. The unhealing wound is featured by sustained expression and elevated levels of pro-inflammatory cytokines such as interleukin-1 (IL-1), interleukin-6 (IL-6), and tumor necrosis factor-α (TNF-α).

To normal wound healing, M1 macrophages dominate for the first three days, after which they translate to M2 macrophages, generally considered as pro-healing cells. However, in diabetic wounds, the transformation of M1 to M2 macrophages is impaired, and M1 macrophages exist for a long time and keep releasing IL-6 and TNF-α, which cause serious tissue damage (8). The impaired transformation is the key factor of the persistent inflammation state in diabetic wound (9).

At the same time, unhealing diabetic wound is often associated with an overload of overwhelming oxidative stress level, which may impair cell function (10). Although moderate amounts of free radicals can promote wound healing, excess oxidative stress will inhibit the migration and proliferation of cells. In general, the balance between reactive oxygen species (ROS) production and elimination is precisely maintained by an intracellular antioxidant protection system. However, excess oxidative stress results in oxidation-reduction imbalance in the diabetic wound microenvironment, beyond the capacity of the endogenous antioxidant system. High oxidative stress level could induce the excessive production of inflammatory factors such as TNF-α and IL-6 and various adhesion molecules by activating the NF-κB signaling pathway (11).

### Impaired angiogenesis

Angiogenesis plays a key role in diabetic wound healing. New blood vessels promote wound healing by delivering nutrients, oxygen and growth factors to the site of injury (12). In order for angiogenesis to occur, endothelial cells must proliferate, stimulating by factors such as VEGF (vascular endothelial growth factor) and FGF (fibroblast growth factor) (13). However, the hyperglycemic environment can lead to dysfunction of vascular endothelial cells, resulting in impaired angiogenesis and reduced capillary density (14). Seitz et al. found VEGF-A in diabetic wounds significantly decreased, being compared to normal ones (15). Meanwhile, vascular endothelial cells in diabetic environment are damaged by oxidative stress, which impairs the function of endothelial cells and induces apoptosis (16). As a result, wound closure is greatly delayed.

### Delayed re-epithelialization and collagen deposition

Wound healing includes inflammatory, proliferative and remodeling phases. During the proliferative phase, a series of changes occur, including re-epithelialization, and ECM deposition (17).

Re-epithelialization aims to restore the epidermal barrier, which relies on the migration of keratinocytes from the wound periphery to center (18). Studies have shown that the expression of ^p125^FAK in keratinocytes is one important factor determining keratinocyte activity. ^P125^FAK is involved in the regulation of cytoskeletal proteins and cell migration. However, high glucose environment prevents phosphorylation of ^p125^FAK, resulting in decreased keratinocyte motility (19). Dedicator of cytokinesis 5 (Dock5) is one of the candidate molecules known to be implicated in protein-protein interactions (20). Recently, Hua Qu et al. found Dock5 regulated keratinocyte migration by promoting ubiquitination of ZEB1 to activate laminin-332/integrin signaling (21).

At the same time, to normal wound healing, it involves epithelial mesenchymal transformation (EMT) in keratinocytes at the wound edge and expression of a series of proteins and molecules. In recent years, it has been found that c-Myc is one of the main proteins regulating EMT of keratinocytes. However, c-Myc in keratinocytes from diabetic wound is overexpressed and the Wnt/β-catenin signaling pathway is activated, leading to the dysfunction of keratinocyte differentiation (22).

Myofibroblast plays a crucial role in the proliferative phase of wound healing, producing collagen and forming the ECM (23). It is known that myofibroblasts are mostly transformed from fibroblasts, characterized by the expression of α smooth muscle actin(α-SMA) (24). The transformation process is regulated by macrophages. M2 macrophages are able to produce growth factors such as insulin growth faction-1 (IGF-1), VEGF and transforming growth faction-β1 (TGF-β1), which stimulate the transformation of fibroblasts to myofibroblasts. However, a high glucose environment prevents the transformation of M1 to M2 macrophages, resulting in decreasing myofibroblasts, inadequate collagen secretion, and delayed wound contraction (25).

Additionally, the obstruction of ECM formation can also affect the re-epithelialization process (26). At the beginning of wound healing, keratinocytes migrate through a temporary matrix formed by type I collagen. This process depends on the balance of matrix metalloproteinases (MMPs) and tissue inhibitor of metalloproteinases (TIMPs). However, in diabetic wounds, the expression of MMPs is increased, while the gene of TIMPs is silenced, intensifying collagen degradation and inhibiting re-epithelialization (3).

## EVs produced by mesenchymal stem cells

EVs are a group of heterogeneous bilayer lipid membrane closed vesicles with different biophysical properties and functions under both physiological and pathological conditions. Exosomes are a class of nanoscale EVs released by a variety of living cells in the body, which can carry information including nucleic acids, proteins and lipids of parental cells. Typically, they range in size from 30 nM to 200 nM. The other two subsets of EVs are microvesicles (100-1000 nM) and apoptotic bodies (500-2000 nM) (27).

Intercellular communication is the transmission of information from one cell to another cell through a medium, which is crucial for the stability of the microenvironment in multicellular organisms. This process is traditionally attributed to the transfer of extracellular proteins and soluble factors between cells. It is novel for exosomes to mediate cell-to-cell communication by delivering biological information molecules, including noncoding RNAs and proteins (28). As an intercellular communication tool, exosomes have several advantages. First, nano-sized exosomes can be easily transferred between cells. Second, the lipid bilayer structure of exosomes provides a protected environment from bioactive molecules being degraded in the extracellular environment. Third, exosomes with specific surface proteins that can be directed to specific organs. These suggest that exosomes are good carriers that can be used to deliver therapeutic drugs, RNAs or proteins to injured tissues (29).

Stem cells have attracted great interest in the research community because of their strong ability to survive and regenerate damaged tissues. Stem cells could be divided into embryonic and adult stem cells (30). Mesenchymal stem cells (MSCs) are one of the most important adult stem cells, characterized by plastic adhesion properties, and differentiation ability. MSCs can be isolated from different sources such as umbilical cord, bone marrow, adipose tissue, skeletal muscle tissue, periodontal ligaments and so on (31). Due to all these properties, MSCs are suitable candidates for tissue repair and regeneration. However, MSCs-based cell therapy has some limitations, such as immune rejection and ectopic tissue formation.

Later observations attributed MSCs’ tissue regenerative ability to their paracrine factors, for their own migration ability to the injury site is low. Exosomes were identified as components of MSC ectocrines and propagated the key regenerative and immunoregulatory characteristics of their parental MSCs (32). Exosomes derived from MSCs (MSCs-exo) have both the advantages associated with exosomes and the characteristics of MSCs. In contrast to their parental MSCs, MSCs-exo has no immunoreactivity, or tumorigenicity. With these properties, MSCs-exo is a promising alternative to MSCs in regenerative medicine (33).

However, it is worth noting that although exosomes have great potential for clinical application, the content of some specific mRNA or protein is very low, or the targeting ability is inefficient, which is ineffective to cure a disease. Therefore, engineered exosomes emerged and have been gradually developed, for example, transfecting exosomes to improve the content of a specific mRNA or modifying them to enhance their targeting ability.

## Mesenchymal stem cell-derived EVs in diabetic wound healing

**Figure 2:**
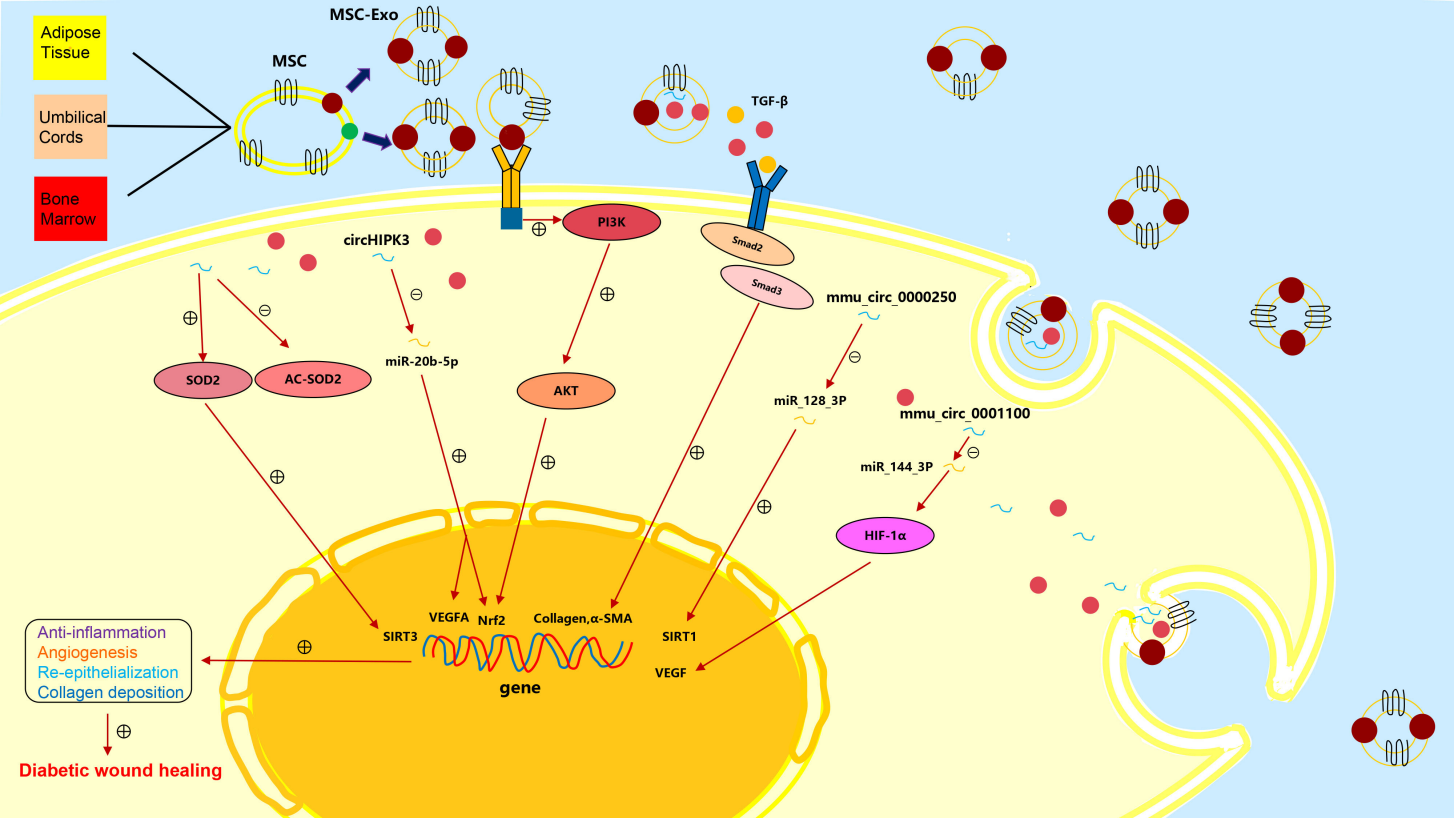
The mechanisms of action of the various exosomes in chronic diabetic wound repair.

Promising therapeutic effects have been achieved by applying MSCs to wound healing (34). As subcellular components of MSCs, exosomes inherit the regenerative and immunoregulatory characteristics of parental MSCs and have special bilayer structure to protect their contents from degradation or damage. MSCs-exo can activate multiple regulation pathways in recipient cells by transporting a variety of bioactive components (proteins, lipids, nucleic acids, etc.) to promote tissue repair and regeneration (35) (**Figure 2**).

### Anti-inflammation

#### Exosomes derived from ADSCs

More and more studies have confirmed that ADSCs-exo is an important medium of intercellular communication, which can reduce inflammatory response and has a good prospect in wound repair. Sen Ren et al. found that ADSCs-exo could secrete eHSP90, which reduced hypoxia and oxidative stress damage to cells (36). Transcription factor nuclear factor-E2 related factor 2 (Nrf2) has a protective effect from oxidative stress and can regulate antioxidant enzyme activity mediated by antioxidant response elements (ARE) (37). In diabetic wound, Nrf2/ARE activity decreased, while oxidative stress levels increased (38). Xue Li et al. have proved that high expression of Nrf2 in ADSCs-exo could reduce ROS, NOX1, NOX4 (the main source of ROS) and inflammatory cytokines in HaCaT cells, fibroblasts and HUVECs (39). SOD is a kind of superoxide scavenger enzyme, which removes harmful superoxide anion free radicals and plays a vital role in maintaining oxidation-reduction balance in cells (40). Yue Zhang et al. found that ADSCs-exo could improve the activity of SOD2, reduce the accumulation of ROS and reactivate endothelial cells by increasing the expression of SIRT3 (41).

#### Exosomes derived from HUCMSCs

Human umbilical cord mesenchymal stem cells (HUCMSCs) are known for their pluripotency. In recent years, HUCMSCs have been used in many clinical fields due to their ease of isolation, purification, and culture (42). HUCMSCs-exo could regulate oxidative stress and reduce cellular hypoxic damage. Chenchen Yan et al. found that HUCMSCs-exo significantly reduced the content of inflammatory factors such as IL-1β, IL-6 and TNF-α, and NOX1 and NOX4 in HUVECs under high glucose environment, reduce oxidative stress, and promote cell proliferation (43) (**Table 1**).

**Table 1:** MSCs-exo in diabetic wound healing.

No.	Study	Year	Exosomes source
1	Sen Ren et al. (36)	2022	ADSCs
2	Xue Li et al. (39)	2018	ADSCs
3	Yue Zhang et al. (41)	2022	ADSCs
4	Chenchen Yan et al. (43)	2022	HUCMSCs

### Promoting angiogenesis

#### Exosomes derived from ADSCs

Many studies have demonstrated that ADSCs-exo can promote angiogenesis. Yue Zhang et al. studied the role of ADSCs-exo in diabetic wound healing using a diabetic mouse model with full-layer back skin injury, and the results showed that angiopoietin 1 (ANG1), fetal liver kinase-1 (FILK1) and VEGF increased after being treated with ADSCs-exo, while endogenous angiogenesis inhibitors angiogenin-1 (VASH1) and thrombopoietin 1 (TSP1) decreased. As a result, the activity of vascular endothelial cells and the potential of angiogenesis enhanced (41). Xue Li et al. found that ADSCs-exo with high Nrf2 were expression had enhanced endothelial progenitor cell proliferation and angiogenesis, through improving the phosphorylation levels of SMP30, VEGF and VEGFR2 (39).

Endogenous MSCs exist in anoxic microenvironments with oxygen concentrations ranging from 1% to 11%, while they are usually isolated and cultured at 21% oxygen concentrations, which may impair the characteristics of MSCs to some extent (44). In fact, hypoxic environment has positive effects on survival and genetic stability of MSCs. Shi Rongfeng et al. found that ADSCs-exo preconditioned by hypoxia could reduce endothelial cell damage in high glucose environment, increase the endothelial progenitor cell differentiation, and ultimately promote diabetic wound healing (45).

#### Exosomes derived from BMSCs

The unbalanced therapeutic effect of exosomes from bone marrow stem cells (BMSCs) and ADSCs on diabetic skin wound healing indicates their different characteristics and clinical potential. Studies have proved ADSCs-exo and BMSCs-exo have distinct biological effects both *in vivo* and *in vitro (* 46). Margherita Pomatto et al. directly compared the therapeutic effects of BMSCs-exo and ADSCs-exo on full-thickness excised skin wounds in diabetic mice, and showed that BMSCs-exo and ADSCs-exo had different effects on chronic wound healing (47). It was found that the molecules carried by ADSCs-exo were mainly related to angiogenesis, while the contents of BMSCs-exo were mainly related to keratinocyte proliferation and viability. ADSCs-exo contained miRNAs that are critical for angiogenesis, such as miR-210 and miR-378 (48). Additionally, some proteins were expressed only in ADSCs-exo, such as Wnt, and angiopoietin1 (Ang1). The Wnt pathway was related to proangiogenic activity (49). Ang1 could restore injured endothelium protein permeability (50). The study showed pro-angiogenic activity of ADSCs-exo could accelerate the diabetic wound healing, whereas the pro-proliferative effect of BMSCs-exo was insufficient to guarantee a therapeutic effect.

#### Exosomes derived from HUCMSCs

In recent years, HUCMSCs have been applied in many clinical fields for their characteristics of easier isolation, purification and culture. Some studies have shown that HUCMSCs can promote the formation of new blood vessels and enhance tissue regeneration (51). The study around the mechanism of HUCMSCs found that HUCMSCs-exo had good stability and immunogenicity, and could transport proteins and growth factors. Chenchen Yan et al. verified that HUCMSCs-exo significantly inhibited HUVECs oxidative stress induced by high glucose, thereby promoting their activity, proliferation and angiogenesis (43). CircRNA is a new class of non-coding RNA, whose 5 'end and 3' end are interconnected, which can regulate the pathogenesis of a variety of diseases. Its complete circular structure makes it resistant to RNA exonuclease degradation and more stable than linear RNA (52). Zunhong Liang et al. found that circHIPK3 in HUCMSCs-exo could inhibit miR-20b-5p and up-regulate Nrf2 and VEGFA (53). At the same time, circHIPK3 overexpression significantly reduced the expression of miR-124 in endothelial cells, thereby protecting endothelial cells from high-glucose damage. These results demonstrated that circHIPK3 regulated the expression of diabetes-related genes by directly binding miRNA. Overexpression of circHIPK3 had a protective effect on cell injury and reduced apoptosis and inflammation to promote angiogenesis.

#### Engineered exosomes

In recent years, it has been found that some non-coding RNAs are abnormally expressed in diabetic wounds and play an important role in the occurrence and development of diabetic wound healing (54). This has prompted scholars to try to regulate the content of non-coding RNAs in diabetic wounds by editing MSCs or exosomes. Rongfeng Shi et al. found that mmu_circ_0000250 had a therapeutic effect to the damaged vascular endothelial cell under high glucose condition so they abstracted exosomes derived from mmu_circ_0000250-modified that ADSCs. It could promote diabetic wound healing by activating autophagy through miR-128-3p/SIRT1 pathway (55). Additionally, they also found ADSCs-exo with high level of mmu_circ_0001100 (circ-Snhg11) promoted diabetic wound healing through activating miR-144-3p/HIF-1α/VEGF signaling pathway (45) (**Table 2**).

**Table 2:** MSCs-exo in promoting angiogenesis.

No.	Study	Year	Exosomes source
1	Yue Zhang et al. (41)	2022	ADSCs
2	Xue Li et al. (39)	2018	ADSCs
3	Rongfeng Shi et al. (55)	2020	Engineered ADSCs
4	Rongfeng Shi et al. (45)	2022	Engineered ADSCs
5	Margherita Pomatto et al. (47)	2021	BMSCs
6	Chenchen Yan et al. (43)	2022	HUCMSCs
7	Zunhong Liang et al. (53)	2022	HUCMSCs

### Accelerating re-epithelialization and collagen deposition

#### Exosomes derived from ADSCs

ADSCs-exo could accelerate re-epithelialization and collagen deposition. Jie Wang et al. found that ADSCs secreted more exosomes under hypoxic environment (hypADSCs-exo), which could promote fibroblast proliferation, migration and secretion of ECM through activating PI3K/AKT pathway. Additionally, 215 miRNAs were up-regulated and 369 miRNAs were down-regulated in hypADSCs-exo compared with ones in ADSCs-exo. Among them, up-regulated miR-21-3p/miR-126-5p/miR-31-5p and down-regulated miR-99b/miR-146-a played important roles in promoting fibroblast proliferation and migration and activating targeted signaling pathways related to immune response (56). The heat-shock protein (HSP) family consists of a group of highly conserved proteins that respond to multiple stresses. Sen Ren et al. demonstrated that ADSCs-exo could secrete eHSP90 by bounding to LRP1, and activating the downstream AKT signaling pathway. As a result, proliferative and migrational abilities of keratinocytes and fibroblasts were promoted (36). Among the complex signaling pathways involving collagen production and tissue fibrosis, TGF-β/Smad3 pathway has been well recognized. TGF-β binds to receptors on fibroblasts, which activates the Smad3 complex to promote nuclear transcription and initiates collagen and α-SMA synthesis. Hsiang-Hao Hsu et al. found that ADSCs-exo could stimulate monocytes or macrophages to secreta more TGF-β1, stimulate the fibroblast proliferation by activating TGF-β/Smad3 signaling pathway, and promote the production of type I collagen in diabetic wounds (57).

#### Engineered exosomes

Studies have shown that miRNAs can be involved in the regulation of homeostasis (58). ADSCs-exo, as a delivery tool for antigen presentation, can load extracellular miRNA particles to prevent them from being hydrolyzed. MiRNA-21 has been shown to be involved in cell proliferation, cell migration, and EMT. Qijun Lv et al. have attempted to combine ADSCs-exo with miR-21-5p, which could up-regulate the expression of MMP-7 through activating Wnt/β-catenin signaling pathway. As a result, keratinocyte migration was significantly enhanced and the re-epitheliization of diabetic wounds was promoted (59) (**Table 3**).

**Table 3:** MSCs-exo in accelerating re-epithelialization and collagen deposition.

No.	Study	Year	Exosomes source
1	Qijun Lv et al. (59)	2020	Engineered ADSCs
2	Jie Wang et al. (56)	2021	ADSCs
3	Sen Ren et al. (36)	2022	ADSCs
4	Hsiang-Hao Hsu et al. (57)	2022	ADSCs

### Application of hydrogels and exosomes composites for diabetic wound

However, the challenge remains in the application of exosomes for wound healing because still exosomes could be cleaned up quickly *in vivo* and the survival time was very short. Therefore, how to prolong the retention time of exosomes in wound without affecting their biological activity has become a research focus.

Multifunctional composite hydrogels based on natural polysaccharides are considered to be ideal dressing for treating diabetic wounds. At the same time, many studies have shown that hydrogels can encapsulate cells, form scaffolds and act as drug carriers to reduce degradation rates (60). Jiayi Yang et al. constructed PF-127 hydrogel and HUCMSCs-exo composite and verified it effectively prolong survival of exosomes when applied to a diabetic skin wound. In addition to its effect of hydrogel-like reducing wound exudates, PF-127 had unique temperature sensitivity (61). PF-127 was liquid at low temperatures and gradually solidified at higher temperatures, meaning it could adapt to the irregular spaces of diabetic wounds. PF-127 has been approved by FDA for human use in USA and is widely used for drug delivery, due to its good biocompatibility and absorbability. In the composite, the biological activity of HUCMSCs-exo was prolonged by PF-127 protection. Due to the continuous release of these exosomes, endothelial cell proliferation and migration were promoted and diabetic wound healing was accelerated. In addition, PF-127 degrades rapidly *in vivo* with no adverse effects on the host. Thus, PF-127 is an ideal biological scaffold for controlling the release of exosomes. Chitosan hydrogel is a hemostatic, antimicrobial, biodegradable and biocompatible carrier for the sustained release of nanoparticles. Xinrong Geng et al. designed a novel MSCs-exo-loaded carboxyethyl chitosan-dialdehyde carboxymethyl cellulose hydrogel for chronic diabetic wound healing (62). The composite synergistically not only enhanced the transition of M1 to M2 macrophages but also promoted angiogenesis by activating VEGF-mediated signalling pathways. Shicong Tao et al. combined chitosan hydrogel with exosomes derived from miRNA-126-overexpressing synovium mesenchymal stem cells (SMSCs). They found the composite could sustainably release exosomes and significantly activate PI3K/AKT and MAPK/ERK pathways, which playing key roles in cell proliferation, migration and angiogenesis (63).

## Conclusions and future perspective

As one of the serious complications of diabetes, diabetic foot ulcer brings massive economic burdens and serious lifestyle challenges to patients. Scientists have devoted much effort to clarifying the pathogenic mechanism of diabetic wound healing and exploiting new therapeutic approaches in recent years. Wound healing is a complicated process involving various cell types and various biochemical factors. Chronic inflammation, impaired angiogenesis, and delayed re-epithelialization and collagen deposition resulted in delayed diabetic wound healing (14). Among the myriad therapeutic approaches to accelerate wound healing, MSCs-exo therapy has attracted increasing attention. MSCs-exo has been proven an ideal nanomaterial to protect their contents, such as microRNAs, lncRNAs, and proteins from the interference of external factors and deliver their contents to target cells to regulate gene expression and function, avoiding the side effects associated with cell therapy, such as immune rejection and tumor formation (6).

However, MSCs-exo could rapidly expand and inactivate at room temperature, which limit its application. Previous studies have shown that various hydrogels can promote healing of chronic wounds (64). Hydrogels can not only enhance cell growth, bone formation and anastomosis but also encapsulate cells, form scaffolds and act as drug carriers (65). MSCs-exo carried in hydrogels showed a reduced degradation rate. The combination of hydrogel and MSCs-exo provides conditions for the application of MSCs-exo in diabetic chronic wound healing. And our future research should focus on the application of MSCs-exo.

Although exosomes provide a new method for treating diabetic wounds, the current research results are mainly from cell or animal experiments. There have been no human trials to prove the clinical value of exosomes, which would be a big leap forward.

In my opinion, our future research should focus on the development of materials containing MSCs-exo and their application in human beings.

## Author contributions

JPZ, JHZ and JH designed the review. XJ, HL, CZ, ZZ and CW consulted literature. MJ and JPZ wrote the manuscript. All authors contributed to the article and approved the submitted version.
